# Shikonin Inhibits Intestinal Calcium-Activated Chloride Channels and Prevents Rotaviral Diarrhea

**DOI:** 10.3389/fphar.2016.00270

**Published:** 2016-08-23

**Authors:** Yu Jiang, Bo Yu, Hong Yang, Tonghui Ma

**Affiliations:** ^1^Liaoning Provincial Key Laboratory of Biotechnology and Drug Discovery, School of Life Sciences, Liaoning Normal UniversityDalian, China; ^2^College of Basic Medical Sciences, Dalian Medical UniversityDalian, China

**Keywords:** CaCC, TMEM16A, inhibitor, shikonin, short-circuit current, rotaviral diarrhea

## Abstract

Secretory diarrhea remains a global health burden and causes major mortality in children. There have been some focuses on antidiarrheal therapies that may reduce fluid losses and intestinal motility in diarrheal diseases. In the present study, we identified shikonin as an inhibitor of TMEM16A chloride channel activity using cell-based fluorescent-quenching assay. The IC_50_ value of shikonin was 6.5 μM. Short-circuit current measurements demonstrated that shikonin inhibited E_act_-induced Cl^-^ current in a dose-dependent manner, with IC_50_ value of 1.5 μM. Short-circuit current measurement showed that shikonin exhibited inhibitory effect against CCh-induced Cl^-^ currents in mouse colonic epithelia but did not affect cytoplasmic Ca^2+^ concentration as well as the other major enterocyte chloride channel conductance regulator. Characterization study found that shikonin inhibited basolateral K^+^ channel activity without affecting Na^+^/K^+^-ATPase activities. *In vivo* studies revealed that shikonin significantly delayed intestinal motility in mice and reduced stool water content in a neonatal mice model of rotaviral diarrhea without affecting the viral infection process *in vivo*. Taken together, the results suggested that shikonin inhibited enterocyte calcium-activated chloride channels, the inhibitory effect was partially through inhbition of basolateral K^+^ channel activity, and shikonin could be a lead compound in the treatment of rotaviral secretory diarrhea.

## Introduction

Secretory diarrhea remains a global health burden and causes major mortality in children under 5 years old (Walker et al., 2013). Repeated diarrhea leads to malnutrition, growth retardation, physical and mental impairment (Petri et al., 2008). Major causes of infectious secretory diarrhea include bacteria such as *Vibrio cholerae* and enterotoxigenic *Escherichia coli*, and viruses such as rotavirus and norovirus as well as some parasites (Walker et al., 2013). Chloride channels located in the luminal membrane of enterocytes represent a significant target for the treatment of secretory diarrhea, because over activation of these channels can cause the massive secretion of chloride ions into the intestinal lumen, and consequently creates an electrochemical gradient and an osmotic force to drive the secretion of sodium and water (Barrett and Keely, 2000). Increased intestinal motility is one of the features in some diarrheal diseases. One of the major chloride channels, cystic fibrosis transmembrane conductance regulator (CFTR), has been shown to be responsible for the enterotoxin-induced secretory diarrhea in cholera and Travelers’ diarrhea (Chao et al., 1994; Grubb, 1995; Thiagarajah et al., 2004; Sonawane et al., 2007). Another enterocyte chloride channel, calcium-activated chloride channels (CaCCs), is thought to be associated with rotavirus-induced and probably other virus-related secretory diarrhea (Morris et al., 1999). TMEM16A was the first CaCC protein that was discovered (Caputo et al., 2008; Schroeder et al., 2008; Yang et al., 2008), and it was found expressed abundantly in the interstitial cells of Cajal (ICC), generating smooth muscle contraction (Huang et al., 2009; Hwang et al., 2009; Ferrera et al., 2010). In rotaviral diarrhea, it is considered that a non-structural protein NSP4 activates TMEM16A/CaCC in the enterocytes (Ball et al., 1996; Morris et al., 1999), but the exact mechanism of this remains to be elucidated.

Apart from TMEM16A, the identities of other gastrointestinal CaCCs (CaCC_GI_) remain unclear. Accumulated evidence show that CaCCs contributes to chronic constipation (Jiang et al., 2015), hypertension (Wang et al., 2015), tumor (Huang et al., 2002; West et al., 2004; Carles et al., 2006), and diarrhea (Ko et al., 2014). Specific inhibitors against CaCCs have been identified, and these have been proven effective at reducing watery stools in a neonatal mice model of secretory diarrhea (Ko et al., 2014). In our previous study, we showed that the anti-diarrheal activity of the traditional Chinese medicine *Bistortae Rhizoma* is due to its inhibition of CFTR- and CaCC-mediated secretion of Cl^-^ ions from intestinal epithelia, and delaying gastrointestinal motility (Yu et al., 2015). There has been some focus on antisecretory therapies aiming at reducing fluid losses and intestinal motility (Donowitz et al., 2012). The present study aimed at identifying new small molecule inhibitors that could inhibit TMEM16A or CaCC_GI_ chloride channels in order to reduce intestinal motility and fluid secretion. This was conducted as part of the effort to search for potentially new therapeutic antidiarrheal drugs.

## Materials and Methods

### Cell Lines, Animals, Virus, and Chemicals

Fischer rat thyroid epithelial (FRT) cells co-expressing TMEM16A and the halide sensor YFP-H148Q/I152L were cultured in F12 coon’s medium (Sigma Chemical Co. St. Louis, MO, USA) supplemented with 10% fetal bovine serum (HyClone company, USA), 100 U/mL penicillin, 100 μg/mL streptomycin and 2 mM L-glutamine. HT-29 cells were cultured in RPMI 1640 medium (Sigma Chemical Co. St. Louis, MO, USA) supplemented with 10% fetal bovine serum (HyClone company, USA), 100 u/mL penicillin, 100 μg/mL streptomycin and 2 mM L-glutamine. Cells were incubated at 37°C in the presence of 5% CO_2_ and 95% humidity air. SA-11 rotavirus was a gift from Professor Verkman in UCSF.

ICR mice (8–10 weeks) were kept with a standard chew diet under specific pathogen-free conditions in a natural day/night circle at Dalian Medical University (Permit Number: SCXK liao 2008-0002). All experiments were carried out in accordance with the Guide for the Care and Use of Laboratory Animals of the National Institutes of Health, and were approved by the Liaoning Normal University Committee on Animal Research.

CFTR_inh_-172 was synthesized as described previously (Garcia et al., 2009). The specific TMEM16A activator E_act_ and CaCC inhibitor CaCC_inh_-A01 were bought from Chembest Research Laboratory Limited (Shanghai). ATP and NaI were purchased from Sangon Biotech (Shanghai) Co., Ltd. Carbachol (CCh) was purchased from EDM Chemicals, Inc. (San Diego, CA, USA). Fluo-4 NW was purchased from Invitrogen (Invitrogen, Carlsbad, CA, USA). Amphotericin B was purchased from Solarbio (Beijing Solarbio Science & Technology Co., Ltd). T16A_inh_-A01 was a generous gift from Professor Verkman at UCSF. Other chemicals, unless indicated otherwise, were all purchased from Sigma (Sigma Chemical Co, St. Louis, MO, USA).

### Iodide Influx Fluorescent Assay

To measure the inhibition of TMEM16A by shikonin, FRT cells expressing TMEM16A were seeded in a black walled clear bottom 96 well plate (Costar, Corning, NY, USA) until confluent. The cells were then washed three times with PBS followed by incubation with different concentrations of shikonin for 10 min. Fluorescence data were recorded with a FLUOstar Galaxy microplate reader (BMG Lab Technologies, Inc.) equipped with HQ 535/30M (535 ± 15 nm) emission and HQ500/20X (500 ± 10 nm) excitation filters (Chroma Technology Corp.), and syringe pumps. Fluorescence was recorded continuously for 14 s, and ATP (200 μM) were pumped into the system along with iodide at 2 s. Iodide influx rates (d[I–]/dt) were computed as described in previous study (Kristidis et al., 1992).

### Intracellular Calcium Measurements

HT-29 cells were seeded in a 96-well black-walled microplate, and incubated until confluent. The cells were washed with PBS three times, and then incubated with Fluo-4 NW according to the manufacturer’s protocol for 15 min. Fluo-4 fluorescence was measured with a FLUOstar Optima fluorescence plate reader equipped with syringe pumps and custom Fluo-4 excitation/emission filters (485/538 nm).

### Short-Circuit Current

Fischer rat thyroid epithelial cells expressing TMEM16A were plated in snapwell inserts and allowed to grow until 95% confluent. Snapwell inserts were mounted in Ussing chambers (Physiological Instruments, San Diego, CA, USA) connected to a VCC MC 6 multi-channel voltage-current clamp via silver/AgCl electrodes with 3 M KCl agar bridges. FRT cell monolayers were stabilized for 30-min in half-Cl^-^ solution (apical, containing in mM: 65 NaCl, 65 Na Gluconate, 2.7 KCl, 1.5 KH_2_PO_4_, 0.5 MgCl_2_, 2 CaCl_2_, 10 Hepes, 10 Glucose, pH 7.4.) and HCO_3_^-^ buffered solution (basolateral, containing in mM: 130 NaCl, 2.7 KCl, 1.5 KH_2_PO_4_, 0.5 MgCl_2_, 2 CaCl_2_, 10 Hepes, 10 Glucose, pH 7.4.). After that, the cells were permeablized basolaterally with amphotericin B (250 mg/L) before activation by E_act_.

Male ICR mice were killed by an overdose of intraperitoneal injection of sodium pentobarbital (100 mg/kg). The colon was removed from each animal and washed with ice-cold modified KH solution (containing in mM: 120 NaCl, 5 KCl, 1 MgCl_2_, 1 CaCl_2_, 10 _D_-glucose, 5 Hepes, 25 NaHCO_3_, 10 μM indomethacin, pH 7.4.). It was then mounted in Ussing Chambers after stripping off the muscularis. The hemi-chambers were each filled with 5 mL KH solution bubbled with 95% O_2_/5% CO_2_ at 37°C. Indomethacin (10 μM) was added into both hemi-chambers to prevent prostaglandin generation. Amiloride (10 μM) was added to the mucosal side to inhibit epithelial Na^+^ current.

For the measurement of apical membrane Cl^-^ current, the basolateral membrane was depolarized with a high potassium concentration solution. After more than 40 min, apical Cl^-^ current was measured in the presence of a serosal-to-mucosal Cl^-^ gradient. The apical solution contains (in mM): 107 *K*-gluconate, 4.5 KCl, 25 NaHCO_3_, 1.8 Na_2_HPO_4_, 0.2 NaH_2_PO_4_, 5.75 Ca-gluconate, 1.0 MgSO_4_ and 12 _D_-glucose. The basolateral solution contains (in mM): 111.5 KCl, 25 NaHCO_3_, 1.8 Na_2_HPO_4_, 0.2 NaH_2_PO_4_, 1.25 CaCl_2_, 1.0 MgSO_4_, and 12 _D_-glucose.

For basolateral K^+^ current measurement, the colonic tissues were placed in a modified KH solution, the apical solution contains (in mM): 116 *K*-gluconate, 4.7 KCl, 5 CaCl_2_, 1.2 MgCl_2_, NaHCO_3_, 1.2 KH_2_PO_4_, 11.1 _D_-glucose. The basolateral solution contains (in mM): 116 Na-gluconate, 4.7 KCl, 5 CaCl_2_, 1.2 MgCl_2_, NaHCO_3_, 1.2 KH_2_PO_4_, 11.1 _D_-glucose. 100 μM ouabain was added to the basolateral solution to inhibit Na^+^–K^+^-ATPase. Subsequent permeabilization of the apical membrane was with nystatin (200 μg/mL).

In the measurement of Na^+^/K^+^-ATPase activity, the mucosal side of the tissue was permeablized with nystatin (200 μg/mL). All cells and tissues were aerated with 95% O_2_/5% CO_2_ at 37°C during the experiments. Transepithelial short-circuit currents were recorded by Acquire and Analyze 2.3 software, transepithelial potential was clamped at 0 mV during the whole processes mentioned above.

### Intestinal Motility Measurement

ICR mice were starved for 24 h and then orally administered shikonin (5.8 μg). After 30 min, 20 mg of 10% activated charcoal diluted in 5% gum arabic was administered orally. After another 30 min, the animals were sacrificed and the small intestines were removed. Peristaltic index was calculated as the ratio of the length that activated charcoal had traveled to the total length of the small intestine.

### Mouse Model of Rotaviral Diarrhea

Neonatal ICR mice (age 4–7 days, weight 2–3 g) were inoculated with 30 μL rotavirus (virus titer 1.2 × 10^7^ pfu/mL) by oral gavage using a polyethylene tube (0.6 mm outer diameter, 0.3 mm inner diameter) and an insulin syringe. The mice were then returned to their mothers and allowed to suckle. Stool samples were collected daily by gentle palpation of the abdomen. In one set of the experiments, the shikonin-treated group received shikonin orally (0.4 and 1.7 μg in 30 μL PBS) the day before virus inoculation, and three times a day until day 3. Control mice received 30 μL PBS alone. The positive control (CaCC_inh_-A01) mice received 34 μg (in 20 μL PBS) CaCC_inh_-A01 by intraperitoneal injection on the day before virus inoculation and twice a day thereafter until day 3. In another set of experiments, shikonin-treated group received shikonin in PBS on the next day of virus inoculation, and three times a day until day 3. Negative control mice received 30 μL PBS alone. The positive control mice received CaCC_inh_-A01 by intraperitoneal injection the day before virus inoculation and twice a day thereafter until day 3.

### Stool Water Content Analysis

To collect the stool samples, the abdomen of the animal was gently palpated. To quantify the water content in the stool, a polymethyl methacrylate (PMMA) slab of 2.0 mm thickness was used in which a 1.0-mm diameter hole was punched to contain a cylindrical 1.57 mm^3^ volume of stool. The cylindrical stool plug was deposited onto a piece of cellulose acetate membrane (saturated with 0.1% neutral red, then dried) and allowed to contact the paper for 3 min in a humidified atmosphere. The wetted area was quantified by digital imaging.

### Histology

Mice were sacrificed on day 3, and the ileum were isolated and rinsed with PBS. Specimens were fixed with formalin for 24 h, and then embedded in paraffin, and stained with haematoxylin and eosin.

### Statistical Analysis

All data were expressed as mean ± SE or as representative traces. One-way or two-way ANOVA followed by Dunnett’s multiple comparison test was used to compare test and control values, and statistically significances were considered at the *p* < 0.05 level.

### Ethics Statement

This study was carried out in accordance with the recommendations of “Guide for the Care and Use of Laboratory Animals of the National Institutes of Health” and were approved by the Liaoning Normal University Committee on Animal Research. All surgery was performed under sodium pentobarbital anesthesia, and possible efforts were made to minimize suffering.

## Results

### Identification of Shikonin as TMEM16A Chloride Channel Inhibitor

**FIGURE 1:**
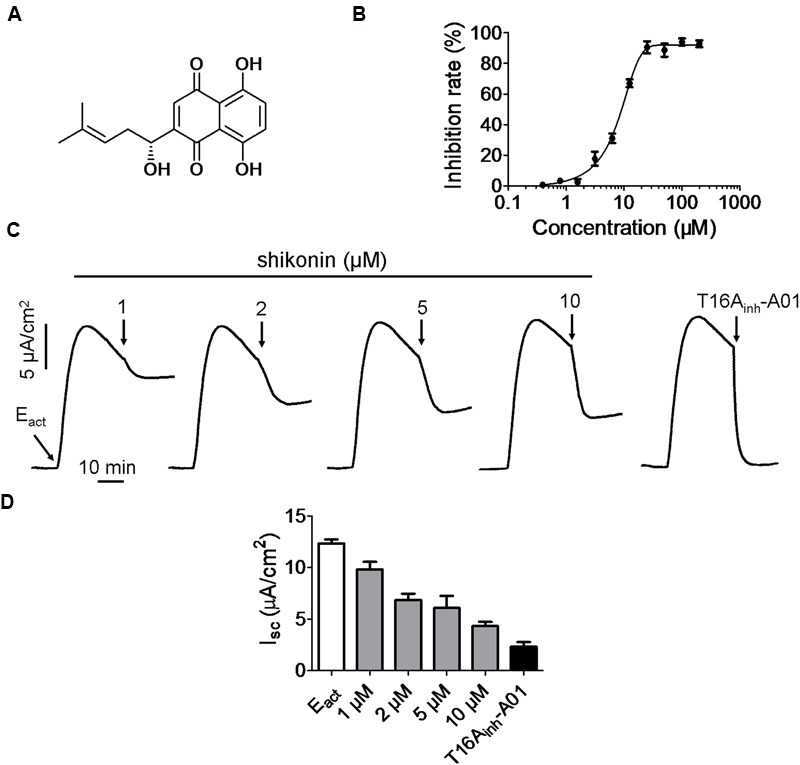
**Inhibition of TMEM16A chloride channel activities by shikonin. (A)** Structure of shikonin. **(B)** Fluorescence quenching assay showing the inhibition of TMEM16A chloride channel activity by different concentrations of shikonin. **(C)** Partial inhibition of E_act_-induced TMEM16A-mediated short-circuit currents by shikonin and T16A_inh_-A01. **(D)** Histogram compares the magnitude of E_act_-induced short-circuit currents inhibited by shikonin and T16A_inh_-A01. The traces shown are typical results from three independent experiments.

TMEM16A was the first CaCC identified, and the effect of shikonin (**Figure 1A**) on TMEM16A chloride channel activity was studied using a previously set up cell-based fluorescent quenching assay. Short-circuit current measurements were performed in FRT cells expressing TMEM16A. Shikonin inhibited ATP-stimulated TMEM16A chloride channel activity with an IC_50_ value of 6.5 μM (**Figure 1B**). Short-circuit current measurement showed that inhibition of TMEM16A chloride currents by shikonin was concentration-dependent, and the highest concentration (10 μM) of shikonin used could only abolish about 65% of the Cl^-^ current stimulated by E_act_ (**Figure 1C**). On the other hand, T16A_inh_-A01 (30 μM) completely inhibited E_act_-induced TMEM16A-mediated short-circuit current (**Figure 1D**).

### Shikonin Inhibits CCh-Induced Short-Circuit Currents in Adult Mouse Colonic Epithelium

**FIGURE 2:**
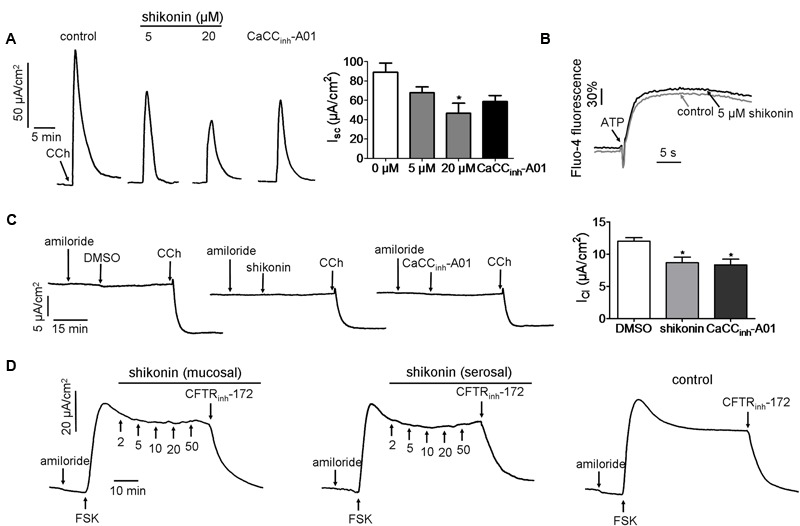
**Effect of shikonin on Cl^-^ transport in mouse colonic epithelium. (A)** Inhibition of CCh-induced short-circuit currents by DMSO, shikonin (5 and 20 μM) and CaCC_inh_-A01 (*n* = 3). The plot compares the magnitude of inhibition seen in the chart. Data are the means ± SEs of three independent tests ^∗^*p* < 0.05. **(B)** Cytoplasmic Ca^2+^ measured by Fluo-4 NW fluorescence under basal conditions following the addition of ATP (100 μM). HT-29 cells were pretreated with DMSO or 5 μM shikonin. **(C)** Inhibition of apical Cl^-^ current by DMSO, shikonin (20 μM) and CaCC_inh_-A01 (50 μM) (*n* = 3). Summary of *I*_Cl_ is shown in the right panel. The plot compares the magnitude of inhibition seen in the chart. Data are the means ± SEs of three independent tests ^∗^*p* < 0.05. **(D)** Effect of shikonin on CFTR chloride channel activity in mouse colonic epithelium. CFTR_inh_-172 was used to abolish FSK-induced short-circuit currents. The traces shown are typical results from three independent experiments.

The efficacy of inhibition exerted by shikonin on endogenous gastrointestinal CaCC (CaCC_GI_) of mouse colon was investigated. Different concentrations of shikonin were added to the mucosal solution, and 15 min later short-circuit currents were stimulated by cholinergic agonist carbachol. Inhibition of CaCC_GI_ current by shikonin was concentration dependent, with 5 and 20 μM shikonin reduced the currents by 24 ± 8.7 and 48 ± 10.7%, respectively (**Figure 2A**), and this was better than the inhibition exerted by 50 μM CaCC_inh_-A01 (34 ± 8.8%).

Since CaCCs can be activated by increases in cytoplasmic calcium concentration, we therefore measured the calcium concentration following activation by ATP. It appeared that ATP-induced cytoplasmic calcium concentration was not affected by 5 μM shikonin (**Figure 2B**).

To exclude the possibility that the reduced transepithelial currents caused by shinkonin were due to inhibition of K^+^ transport, transepithelial currents were measured under depolarization condition. Experiments were performed under the condition that the basolateral membrane was depolarized by high concentration of K^+^. The results indicated that apical application of shikonin (20 μM) and the CaCC specific inhibitor CaCC_inh_-A01 (50 μM) still could reduce CCh-induced short-circuit currents by 27.8 ± 2.7 and 30.5 ± 3.3%, respectively, as compared to the DMSO control (**Figure 2C**), suggesting that shikonin directly inhibited chloride currents.

The efficacy of shikonin-mediated inhibition of CFTR chloride channel activity was also tested *ex vivo* in freshly isolated mouse colonic mucosa. Short-circuit currents were stimulated by FSK (20 μM), and then shikonin (2–50 μM) was added into the mucosal or serosal solution to investigate the inhibitory effect of shikonin on FSK-stimulated CFTR-mediated Cl^-^ current. Shikonin did not affect CFTR-mediated short-circuit currents in mouse colonic epithelia (**Figure 2D**). The remaining currents were abolished by CFTR_inh_-172 (100 μM). These results suggested that shikonin directly inhibited CaCC_GI_ chloride activity in mouse colonic epithelia.

### Characterization of Shikonin-Inhibited CaCC Currents

**FIGURE 3:**
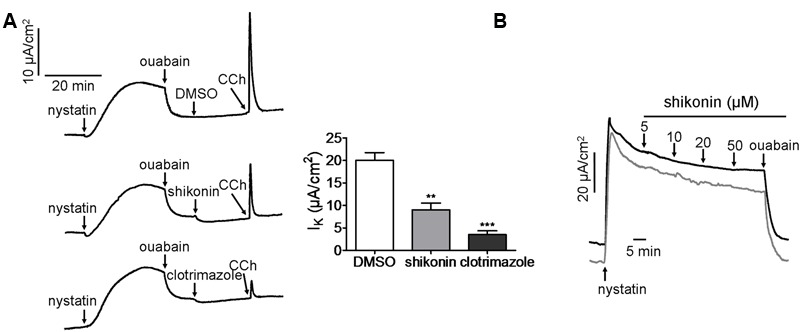
**Characterization of shikonin-inhibited CaCC currents. (A)** Inhibition of basolateral K^+^ channel by DMSO, shikonin (20 μM) and clotrimazole (50 μM). Summarized *I*_K_ is shown in the right panel. The plot compares the magnitude of inhibition seen in the chart. Data are the means ± SEs of three independent tests ^∗∗^*p* < 0.01, ^∗∗∗^*p* < 0.0005. **(B)** Effect of shikonin on Na^+^/K^+^-ATPase in mouse colonic epithelium. Short-circuit current was induced by intracellualr Na^+^ loading following mucosal permeabilization by nystatin (200 μg/mL). The traces shown are typical results from three independent experiments.

Apical secretion of Cl^-^ in the enterocyte requires the driving force provided by Na^+^/K^+^-ATPase, Na^+^/K^+^/2Cl^-^ cotransporter and K^+^ channels located in the basolateral membrane. Thus inhibition of these transporters will indirectly lead to reduced apical chloride secretion in the enterocyte. We therefore analyzed the effect of shikonin on basolateral K^+^ channel and Na^+^/K^+^-ATPase in mouse colonic epithelia. As seen in **Figure 3A**, after permibilization of apical membrane by nystatin, shikonin decreased CCh-stimulated short-circuit current by 55.0 ± 4.3% compared to DMSO control, whereas the basolateral K^+^ channel inhibitor clotrimazole decreased the current by 82.5 ± 2.8%, suggesting that shikonin significantly inhibited Ca^2+^-activated basolateral K^+^ channel. To measure the activity of Na^+^/K^+^-ATPase, short-circuit current was analyzed after mucosal permeabilization and addition of different concentrations (5–50 μM) of shikonin to the serosal solution. As shown in **Figure 3B**, shikonin had no effect on Na^+^/K^+^-ATPase-mediated short-circuit current, and the nystatin-stimulated currents were completely abolished by the Na^+^/K^+^-ATPase inhibitor ouabain (500 μM), suggesting that inhibition of chloride transport by shikonin was not via inhibition of basolateral Na^+^/K^+^-ATPase activities.

### Inhibition of Adult Mouse Intestinal Motility by Shikonin

**FIGURE 4:**
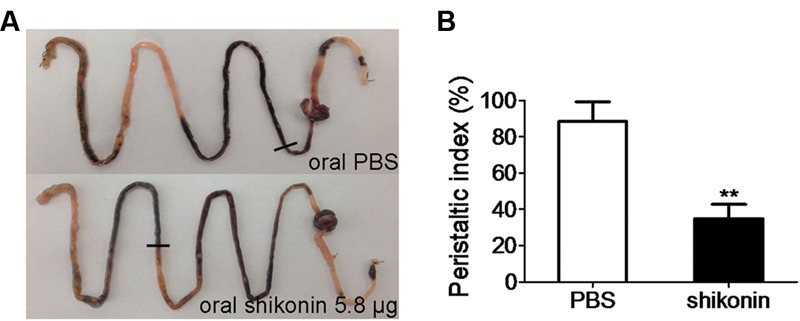
**Inhibition of gastrointestinal motility by shikonin. (A)** Photographs of isolated mouse intestinal tracts showing the distance traveled by activated charcoal after administration of PBS or shikonin by oral gavage. **(B)** Comparison of the peristaltic indexes (distances activated carbon moved in the intestines) between PBS- and shikonin-treated animals. Data are the means ± SEs of three independent experiments. ^∗∗^Significantly different from PBS-treated animal at the *p* < 0.01 level.

Enhanced intestinal motility is also a symptom in some infectious diarrhea. TMEM16A is expressed abundantly in the ICC where it generates smooth muscle contraction, thereby mediates gastrointestinal motility (Huang et al., 2009; Hwang et al., 2009; Ferrera et al., 2010). Therefore the effect of shikonin on gastrointestinal motility was investigated (**Figure 4A**). Oral administration of 5.8 μg shikonin significantly reduced intestinal peristalsis and delayed charcoal movement in mice, yielding a peristaltic index of 34.9 ± 9.1%, compared to 82.3 ± 2.9% induced by PBS (**Figure 4B**).

### Shikonin Prevents Watery Stools in Rotavirus Infected Neonatal Mice

**FIGURE 5:**
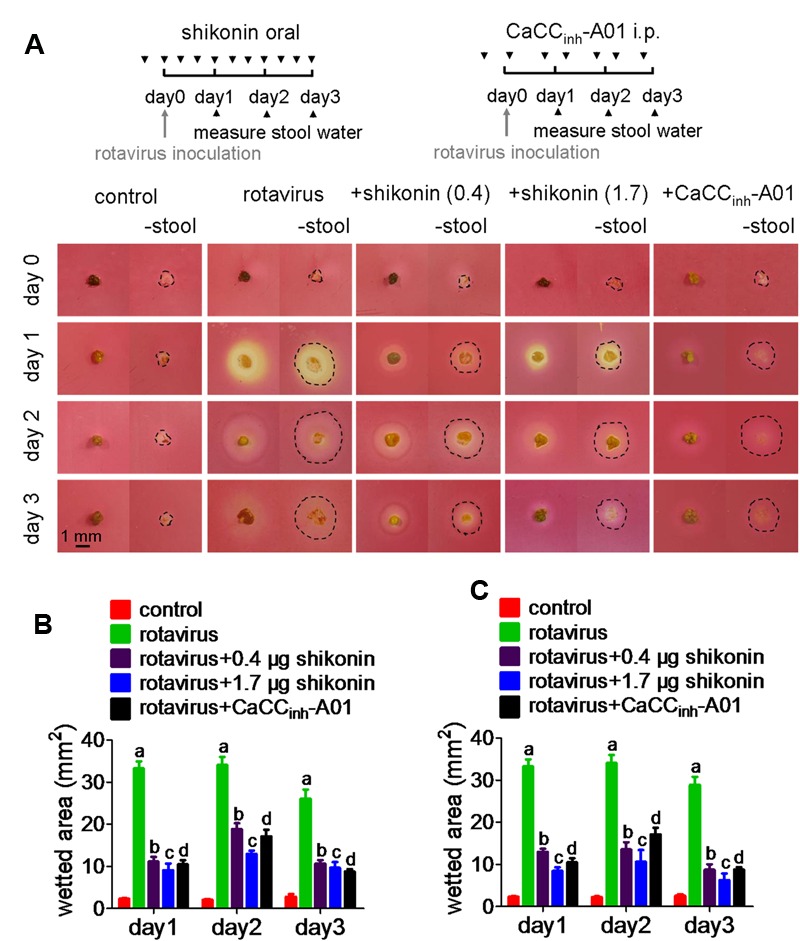
**Effect of shikonin on rotaviral diarrhea in a neonatal mouse model. (A)** Schematic illustration of the protocol used to induce rotaviral diarrhea in mice and the image of the stools obtained from these animals. Mice were orally given a single dose of shikonin (0.4 and 1.7 μg) the day before rotavirus inoculation, and three doses per day for the next 4 days. Positive control mice were intraperitoneal injected with a single dose (34 μg) of CaCC_inh_-A01 the day before rotavirus inoculation followed by two doses per day for the next 4 days. Photographs of the cellulose acetate membranes taken 3 min after deposition of the stool specimens (left) and after removal of the stool specimens (right). Wetted area is demarcated by the broken line. **(B)** The plots compare the size of the wetted area on the cellulose acetate membrane after removal of the stools. **(C)** Similar study as in **(A)**, except that shikonin gavage started on day 1(SE, *n* = 4–6 mice). Data are the means ± SEs of three independent experiments. Significant difference between rotavius-inoculated group (a) and shikonin-(b and c) or CaCC_inh_-A01 (d)-treated groups (*p* < 0.0005).

The inhibitory effect of shikonin on rotaviral diarrhea *in vivo* was tested with two protocols as described above. **Figure 5A** (top) shows the first protocol used to induce rotaviral diarrhea in neonatal mice. Grossly watery stools started on day 1 after rotavirus inoculation, and it lasted for the next 3 days. Comparison of the images of the cellulose acetate membrane taken 3 min after the deposition of the stools with those taken after the removal of the stools showed the wetted area (marked by thin broken circular lines) varied among the different treatments over the 3 days (**Figure 5A** bottom). The water content decreased in shikonin- and CaCC_inh_-A01-treated groups, as shown by the smaller wetted area. **Figure 5B** compares the wetted area left by the stools on days 1–3 from mice treated with shikonin or CaCC_inh_-A01. In the second protocol, shikonin was also effective when administered on day 1, after watery diarrhea had begun, with significantly lower water content in the stools on days 2–3 (**Figure 5C**). These results demonstrated that shikonin could prevent water diarrhea following rotavirus inoculation in neonatal mice, and the effect was better than CaCC_inh_-A01.

### Shikonin Does Not Inhibit Rotavirus Infection

**FIGURE 6:**
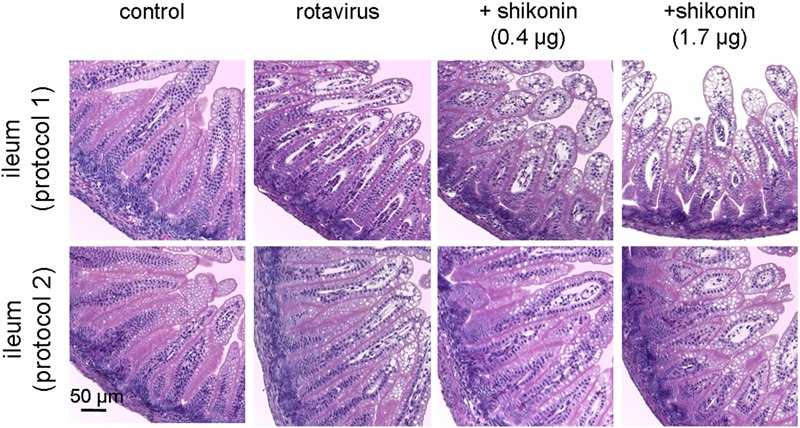
**Effect of shikonin on rotavirus infection in neonatal mice model.** Histological analysis of mouse ileum on day 3 of the infection. Images shown are photographs taken of paraffin-embedded sections from control mice, rotavirus-inoculated control and shikonin-treated mice.

Rotavirus infection of the intestine was further verified by histology, which revealed marked vacuolization of enterocytes in the infected animals. Three days after infection, villus tip swelling and large vacuoles were seen in enterocytes lining in the surface of villi in the rotavirus-infected groups (**Figure 6**). Vacuoles were not seen in the non-infected mice. Shikonin-treated mice also showed vacuolization similar to that in the untreated rotavirus-infected mice, supporting the conclusion that shikonin reduced water diarrhea through antisecretory action rather than preventing rotavirus infection.

## Discussion

Viral diarrhea remains one of the most fatal diseases in children in both developed and developing countries. The present study found that shikonin inhibited TMEM16A and CaCC_GI_ in the intestine, and significantly reduced intestinal motility in mice and stool water content in a neonatal mice model following rotavirus infection. Shikonin was inhibitory against CaCC-mediated colonic epithelial Cl^-^ transport in isolated mouse colon. Unlike tannic acid and gallotannins, which also inhibits another major enterocyte chloride channel, CFTR or ENaC (Namkung et al., 2010), shikonin was not inhibitory toward CFTR or Na^+^/K^+^-ATPase, thus displaying stronger selectivity for the inhibition of CaCC chloride channel activity than tannic acid and gallotannins. However, it is important to note that neither shikonin nor CaCC_inh_-A01 could completely inhibit CCh-stimulated short-circuit currents in isolated mouse colon. Basolateral Ca^2+^-activated K^+^ channels have been shown to express in human and mouse colon (Rufo et al., 1996, 1997; Sandle and Rajendran, 2012). Ourt study didn’t ignore the possibility of indirect inhibitory effect of shikonin via basolateral K^+^ channel. As assumed, basolateral Ca^2+^-activated K^+^ channel did account, at least a part, for the inhibition of CCh-induced chloride current by shikonin.

Reduction of stool water content in rotaviral neonatal mice by shikonin observed in this study probably occurred via an anti-secretory action of shikonin, which involved inhibition of CaCC_GI_ chloride channel activity. Although TMEM16A exists in enterocytes, some investigators have proposed that secretory diarrhea caused by the rotaviral non-structural protein NSP4 is predominantly through the activation of epithelial TMEM16A in the intestine. One study that used the small molecule TMEM16A inhibitor T16A_inh_-A01 has demonstrated that TMEM16A constitutes only a minor component of the intestinal epithelial CaCC (Namkung et al., 2011). Our previous work has shown that TMEM16A and CaCC_GI_ have different characteristics, since the lignan compounds kobusin and eudesmin can affect TMEM16A and CaCC_GI_ differently, inhibiting TMEM16A, while activating CaCC_GI_, (Jiang et al., 2015). We have shown in this study that shikonin was inhibitory toward CaCC_GI_-mediated short-circuit currents in both cell culture model and isolated mouse colon. In addition, *in vivo* studies showed that shikonin reduced water content in a neonatal mice diarrhea model without affecting the rotavirus infection process (**Figure 6**). These findings supported the view that the primary pathway of watery diarrhea associated with rotaviral infection is through activation of CaCC_GI_ rather than TMEM16A by NSP4, thereby enhancing the accumulation of fluid. Furthermore, inhibition of TMEM16A by shikonin delayed gastrointestinal motility, helping to prolong the fluid absorption time to further decrease net fluid secretion.

As the major component of Zicao, the dried root of *Lithospermum erythrorhizon*, shikonin has been broadly used due to its anti-inflammatory activity (Chen et al., 2002). Shikonin has been reported to have antioxidant, antibacterial, antiparasitic, antiviral and wound-healing activities (Andujar et al., 2013). Shikonin might be used in the treatment of asthma. Takano-Ohmuro et al. (2008) has explored the use of shikonin in asthma by focusing on its anti-inflammatory activity (Takano-Ohmuro et al., 2008). Other investigators have used a mouse asthma model to demonstrate that shikonin inhibits bone marrow-derived dendritic cell maturation *in vitro*, and allergic action as well as tracheal hyperresponsiveness *in vivo* (Lee et al., 2010). Since TMEM16A is expressed in airway smooth muscle cells and it participates in smooth muscle contraction (Huang et al., 2012), we assumed that TMEM16A may be involved in shikonin-mediated inhibition of asthma. In our study, shikonin was found to inhibit TMEM16A chloride channel activity, indicating that shikonin might alleviate asthma by inhibiting smooth muscle contraction in the trachea.

Despite the many positive benefits of shikonin, it is not without toxicity. Intraperitonal injection of shikonin has been demonstrated to result in some toxicity, with an LD_50_ of 20 mg/kg (Sankawa et al., 1977). Pharmacokinetics study has shown that shikonin absorption is fast if given by oral gavage and muscle injection, as it is barely detected in the plasma after 1 min, with a oral gavage yielding a bioavailability of about 34% (Wang et al., 1988). In this study, the doses used for intestinal motility (0.38 mg/kg) and rotaviral infection in the mice model (0.69 mg/kg) were below the doses that are considered as toxic.

## Conclusion

The present study revealed shikonin as a new inhibitor against TMEM16A and CaCC_GI_ chloride channel, adding to the list of biological activities that this compound already possesses, and showed that shikonin could be leading drug in the treatment of rotaviral diarrhea.

## Author Contributions

YJ carried out the experimental work and the data collection and drafted the manuscript. BY participated in the design and coordination of experimental work, and acquisition of data. HY and TM carried out the study design and revised it critically for important intellectual content. All authors read and approved the final manuscript.

## Conflict of Interest Statement

The authors declare that the research was conducted in the absence of any commercial or financial relationships that could be construed as a potential conflict of interest.
